# Disruption of *GRR1* in *Saccharomyces cerevisiae* rescues *tps1Δ* growth on fermentable carbon sources

**DOI:** 10.17912/micropub.biology.000927

**Published:** 2023-08-03

**Authors:** Anqi Chen, Patrick A. Gibney

**Affiliations:** 1 Department of Food Science, Cornell University, Ithaca, New York, United States; 2 Science Center for Future Foods, Jiangnan University, Wuxi, Jiangsu, China

## Abstract

In *Saccharomyces cerevisiae* , trehalose-6-phosphate synthase (Tps1) catalyzes the formation of trehalose-6-phophate in trehalose synthesis. Deletion of the *TPS1* gene is associated with phenotypes including inability to grow on fermentable carbon sources, survive at elevated temperatures, or sporulate. To further understand these pleiotropic phenotypes, we conducted a genetic suppressor screen and identified a novel suppressor, *grr1* Δ, able to restore *tps1* Δ growth on rapidly fermentable sugars. However, disruption of *GRR1* did not rescue *tps1* Δ thermosensitivity. These results support the model that trehalose metabolism has important roles in regulating glucose sensing and signaling in addition to regulating stress resistance.

**Figure 1. Deletion of GRR1 suppresses tps1 Δ inability to grow on glucose or fructose:**
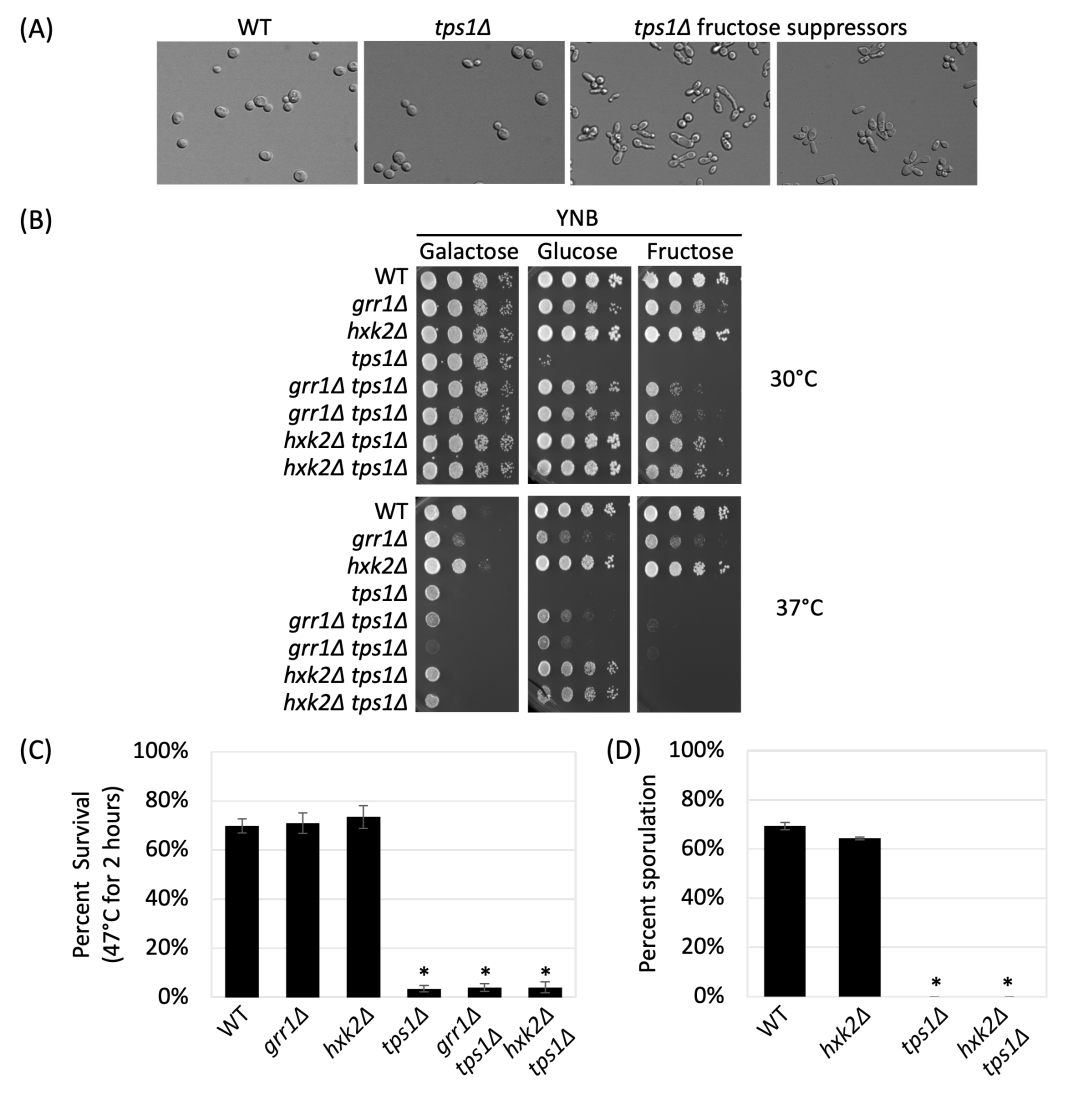
**(A) Suppressor morphology** - Cells from indicated strains were collected from colonies on YNB+fructose plates, then photographed using standard brightfield microscopy with a 100X oil immersion objective; **(B) Carbon source utilization** - Indicated strains were grown overnight in YNB+galactose liquid medium before 10-fold serial dilutions were prepared and spotted onto the indicated media. The initial dilution had an OD _600_ of 1.0. Listed carbon sources were present at 2% (w/v). Plates were incubated at 30°C for 3 days and at 37°C for 4 days. Duplicate strains were derived from independent cultures and represent biological duplicates; **(C) Thermotolerance** - Indicated strains were grown overnight in YNB+galactose at 30°C and were then diluted into YNB+galactose to an OD _600_ = 0.5 before growing for another 24 hours to stationary phase. An aliquot of culture was then heat shocked in a 47°C thermomixer for 2 hours. Dilutions of both pre- and post-heat shocked cells were plated on rich media containing galactose (YPGal) and incubated at 30°C for 2 days to measure viability by counting colony forming units; **(D) Sporulation efficiency** - Indicated strains were grown to log phase in YPGal, sporulated in 1% potassium acetate, and incubated at room temperature on a roller wheel for 6 days before measuring percent sporulation by counting at least 300 cells. Sporulation efficiency was calculated as the proportion of observed tetrads compared to the total number of observed cells. For panels (A), (B), and (C) all strains are haploid. For panel (D), all strains are diploid and deletion strains are homozygous for each listed deletion. Asterisks represent statistical difference (p<0.05) between the mutants and the wild type. At least three independent biological replicates were performed for each phenotype test.

## Description

Trehalose is a non-reducing disaccharide of two glucose units present in many living organisms (Elbein et al., 2003) . In the yeast *Saccharomyces cerevisiae* , trehalose-6-phosphate synthase (Tps1) catalyzes the first step of trehalose synthesis, producing trehalose-6-phosphate (T6P), which is then dephosphorylated by trehalose-6-phosphate phosphatase to produce trehalose (Chen & Gibney, 2022) . Loss-of-function *TPS1* mutants in *S. cerevisiae* have been studied extensively in a variety of laboratory and wild strains and a panel of phenotypes have been reported (Chen et al., 2022; Gancedo & Flores, 2004) . In addition to a lack of metabolic ability to synthesize T6P or trehalose, *tps1* mutants exhibit other unrelated physiological defects, such as inability to utilize fermentable carbon sources, sporulation deficiency, high sensitivity to elevated temperature and alterations in glycogen levels (Gibney et al., 2015; Hohmann et al., 1993; Hottiger et al., 1987; van Aelst et al., 1993) .

Tps1 plays a key role in carbon and energy homeostasis in yeast, as shown by the well-documented loss of ATP and hyperaccumulation of sugar phosphates in response to glucose addition in *tps1Δ* (Vicente et al., 2018) . However, a mechanistic understanding regarding the inability of *tps1* mutant to cope with fermentable sugars is still a matter of debate. In this work, we conducted a genetic suppressor screen to identify genes important for *tps1Δ* to grow on fructose, one of the commonly consumed fermentable carbon sources in *S. cerevisiae* . Among the 13 independent suppressors isolated, we found 2 exhibited normal cellular morphology whereas 11 were abnormal ( Figure 1A ). Whole genome sequencing revealed that those with normal morphology had mutations in *HXK2* , which encodes the major fermentative hexokinase enzyme, including a 1 bp deletion resulting in a frameshift at proline-6 and a 3 bp deletion that removes the highly conserved valine-188 amino acid. Loss-of-function *HXK2* mutations act as genetic suppressors of *tps1Δ* growth on fermentable carbon sources in multiple previous studies (Deroover et al., 2016; Hohmann et al., 1993, 1999) . Besides *hxk2* , we found that the 11 isolates conferring to abnormal cellular morphology all had mutations in *GRR1* , a novel suppressor of *tps1Δ* not previously reported. Observed mutations in *GRR1* appeared to be loss-of-function mutations (5/11 mutations were nonsense mutations, introducing stop codons at E157, E174, S241, S734, and S772; 5/11 mutations were frameshifts resulting from 1 bp deletions at I166, F605, N585, F605, and V942; 1 of the 11 mutations was a deletion spanning amino acids 419-461).

Grr1 is a member of the SCF-ubiquitin ligase complex, a central component of the glucose sensing/signaling network responsible for glucose-induced gene expression in *S. cerevisiae* (Bailey & Woodword, 1984; Erickson & Johnston, 1994; F. N. Li & Johnston, 1997; Vallier et al., 1994) . Loss-of-function mutations in *GRR1* were found to relieve repression of many glucose-repressed genes and prevent glucose induction of several *HXT* genes encoding glucose transporters (Bailey & Woodword, 1984; Flick & Johnston, 1991; Ozcan & Johnston, 1995) . Genetic analysis suggested that *GRR1* acts at an early stage of glucose signal transduction to inhibit the function of Rgt1, a transcriptional repressor of hexose transport genes, thereby causing de-repression of *HXT* gene expression (Erickson & Johnston, 1994; Flick & Johnston, 1991; Vallier et al., 1994) . In addition to defective glucose signaling, *grr1* mutants also exhibit a severe change in cell morphology, characterized by elongated sausage-shaped cells and buds, due to failed degradation of the G1 cyclins Cln1 and Cln2, which explains the morphology observed among *grr1* suppressors ( Figure 1A ) (Bailey & Woodword, 1984; Conklin et al., 1993; Flick & Johnston, 1991; Vallier & Carlson, 1991) .

*HXK2* , on the other hand, has been identified as a bi-functional enzyme, being both a catalyst for phosphorylation of glucose, fructose, and mannose in the cytosol and an important regular of glucose repression by binding with Mig1 as a heterodimeric transcriptional repressor in the nucleus (Vega et al., 2016) . One possible explanation for *tps1* inability to grow on rapidly fermentable carbon sources is loss of control exerted over hexokinases by T6P (Blázquez et al., 1993; Hohmann et al., 1996). An excessive flux of glucose through upper glycolysis cannot be accommodated by lower glycolysis, causing an accumulation of metabolites and a depletion of ATP (González et al., 1992; Navon et al., 1979). However, there are several indications that the absence of T6P inhibition of hexokinase alone is not sufficient to explain the glucose-induced defects in the *tps1* mutant (Blázquez & Gancedo, 1994; Bonini et al., 2000, 2003; Hohmann et al., 1996; Walther et al., 2013). To confirm whether loss-of-function mutations in *GRR1* can suppress the carbon source defects of *tps1Δ* , we independently generated a complete deletion of the *GRR1* gene. For comparison, we included a deletion of *HXK2* as a previously observed suppressor. As shown in Figure 1B, all tested mutants grew well on media containing respiratory carbon sources galactose (preferred respiration) when incubated at 30°C. However, *tps1Δ* growth on glucose and fructose was severely compromised. The small sub-population of *tps1Δ* able to grow on glucose has been described in previous studies (Gibney et al., 2020; van Heerden et al., 2014) . As expected, deletion of *HXK2* restored *tps1Δ* growth defect on fructose and glucose ( Figure 1B ) (Blázquez et al., 1993). We also confirmed that *grr1Δ* rescued *tps1Δ* growth on both glucose and fructose ( Figure 1B ), though not fully to wild type levels of growth. *grr1Δ* mutants were reported to form smaller colonies than wild type cells, which we observe on glucose and fructose but not galactose. It is possible that slow growth in these conditions can partially explain the incomplete suppression observed for *grr1Δtps1Δ* (Flick & Johnston, 1991) .

Heat sensitivity of *tps1Δ* strains has been reported in a number of previous studies (Eleutherio et al., 1993; Gibney et al., 2015; Hottiger et al., 1989; Singer & Lindquist, 1998) . Here, we examined the ability of these mutants to grow at elevated temperature, 37°C. The viability of *tps1Δ* was noticeably reduced at this slightly raised temperature, but deletion of *GRR1* or *HXK2* did not to rescue this phenotype, suggesting the *tps1Δ* carbon-source utilization defect suppressed by deletion of either *GRR1* or *HXK2* is independent of the Tps1 function related to growth at elevated temperatures ( Figure 1B ). Beyond growth at elevated temperatures, we also examined thermotolerance, the ability of cells to survive when treated with a lethal heat dosage for a short period of time. To understand whether disruption of *GRR1* affects *tps1Δ* thermosensitivity, stationary phase cells were heat shocked at 47°C for 2 hours. Wild type, *grr1Δ* , and *hxk2Δ* mutants were able to maintain over 60% survival after heat shock, while the viability of *tps1Δ* , *grr1Δtps1Δ* , and *hxk2Δtps1Δ* dropped significantly ( Figure 1C ). Therefore, although both *grr1Δ* and *hxk2Δ* are suppressors of *tps1Δ* failure to grow in glucose and fructose, these mutations do not restore *tps1Δ* thermotolerance to the wild type levels, again suggesting an independent role for Tps1 in temperature stress phenotypes.

Another phenotype of associated with *TPS1* deletion mutants is failure to sporulate (de Silva-Udawatta & Cannon, 2001; Gibney et al., 2015; Liu et al., 2020). As *grr1Δ* mutants have abnormal cell morphology, we were unable to easily perform mating, tetrad dissection, or observe/quantify sporulation for these mutants. We were therefore not able to examine sporulation of homozygous diploid *grr1Δ* and *grr1Δtps1Δ* strains. However, we were still able to examine whether *hxk2Δ* can suppress the *tps1Δ* sporulation defect. Similar to the heat stress phenotypes, the *hxk2Δ tps1Δ* strain did not sporulate ( Figure 1D ). Again, failure to suppress the *tps1Δ* sporulation phenotype with *hxk2Δ* indicates that the role of Tps1 in sporulation is likely independent from the role of Tps1 in glucose sensing and signaling.

Taken together, we present evidence that disruption of *GRR1* is a novel suppressor of *tps1Δ* failure to grow on rapidly fermentable sugars, including glucose and fructose. Two previously identified suppressors of the *tps1Δ* carbon source utilization phenotype, *hxk2Δ* and *snf4Δ* , are found in the Snf1 branch of the glucose sensing and signaling network (Blázquez & Gancedo, 1995; Kim et al., 2013; Moreno et al., 2005). Deletion of *GRR1* is the first identified suppressor from the Snf3/Rgt2 branch of glucose sensing and signaling, potentially suggesting a more general role for trehalose metabolism in regulating carbon source utilization (Kim et al., 2013; Moreno et al., 2005) . Proper control of glucose influx to glycolysis is required for a wide range of glucose-signaling effects in yeast, as was demonstrated with the *tps1Δ* mutant showing severe deregulation of glycolysis after addition of glucose (Thevelein & Hohmann, 1995; van Vaeck et al., 2001) . The relationship between the glucose-related defects of the *tps1Δ* mutants and the function of Tps1 remains unclear, though a number of models have been proposed. There are two main possibilities: either the Tps1 protein has a separate function as a free protein in the mechanism of glucose sensing or there is a close interaction between the trehalose-synthesizing system and the glucose sensing/signaling system (van Aelst et al., 1993) . Further studies combining both genetic and biochemical approaches could increase our understanding of the composition and functional interactions within the glucose sensing and signaling network, including the role of trehalose metabolism.

## Methods

Yeast media and growth

Yeast cell growth and standard laboratory manipulation were performed as described (Guthrie & Fink, 1991). All media used were either minimal (YNB: 0.67% w/v yeast nitrogen base without amino acids plus 2% w/v indicated carbon sources), or rich (YP: 2% w/v Bacto peptone, 1% w/v yeast extract, 2% w/v indicated carbon sources). Solid media formulations included 2% w/v agar and were poured into standard 10cm plastic Petri dishes (Petri, 1887) . Measurements of cell density were performed by measuring absorbance at 600nm using a Gensys 6 UV-Vis spectrophotometer (Thermo Fisher). For comparative growth assays, cells were spotted onto relevant solid growth media. Cell spotting was performed by dilution of a stationary phase culture to an initial OD _600_ of 1.0, followed by 10-fold serial dilutions. All dilutions were then spotted onto solid media using a Replica Plater for 96-well Plate, 8 x 6 array (Sigma-Aldrich). Pates were incubated at indicated temperatures and times as noted in the figures and legends. At least three independent biological replicates were performed on different days for spotting assays shown in figures, and a representative image is shown.

Isolation of suppressor of *tps1* defective growth on fructose

Independent cultures of *tps1Δ* cells were started from 20 separate colonies and grown overnight in liquid rich media containing 2% galactose (YPGal) at 30°C *.* The entire culture volume was then collected and washed with water once before being plated onto solid minimal medium containing 2% fructose (SF). The plates were incubated at 30°C for 3 days before they were examined for growth. Suppressor colonies were evident on 13 of the fructose-containing plates, and a representative colony from each plate was isolated. These 13 independent suppressors were selected for whole genome sequencing, along with tps1 *Δ* strain for comparison. Genomic DNA libraries were prepared using the TruSeq DNA library prep kit (Illumina) and sequenced using an Illumina HiSeq 2500 to an average coverage depth of 200X. Illumina adapters were trimmed from the reads by Trimmomatic, and the reads were aligned to the *S. cerevisiae* reference genome (R64.2.1; www.yeastgenome.org) using BWA-MEM with default options selected (Bolger et al., 2014; H. Li, 2013) . Potential suppressor mutations, including SNPs, amplifications, and indels, were identified through manual examination of the variant call format (vcf) files produced after read alignment along with visualization of the alignments using Integrated Genome Viewer (Robinson et al., 2011) . As most identified mutations were predicted to cause loss-of-function, both identified suppressor genes were confirmed through independent construction and testing of complete gene deletions mutants.

Assessment of thermotolerance

To assess thermotolerance, minimal medium containing 2% galactose (SGal) was inoculated with a single colony and grown overnight. Cells were then diluted into the same minimal medium to an OD _600_ = 0.05 and grown another 24 hours to stationary phase. Two aliquots of 0.8 mL cell culture were removed into separate microcentrifuge tubes. For the heat shock, one of the aliquots was incubated in a 47°C thermomixer for 2 hours. Both pre- and post-heat shocked cell dilutions were plated on rich media containing galactose (YPGal) and incubated at 30°C for 2-3 days to measure viability by counting colony forming units. At least three independent biological replicates were performed for each thermotolerance assay.

Measurement of sporulation efficiency

Sporulation was performed by growing cells to log phase in YPGal, collecting cells by centrifugation, washing twice in 1% potassium acetate, then resuspending in 1% potassium acetate. Cells were then incubated at room temperature on a roller wheel for at least 6 days before evaluating percent sporulation by counting at least 300 cells. Sporulation efficiency was calculated as the proportion of observed tetrads compared to the total number of observed cells. At least three independent biological replicates were performed for each sporulation efficiency assay.

Statistical analysis

All experiments were conducted using at least three independent biological replicates. Mutants were evaluated for statistical significance compared to the wild type strains using a paired *t* -test and presented as the mean and standard deviation. The asterisks (*) indicate the mutant phenotype showed a difference ( *p* < 0.05) compared to the wild type ( *p* -values were not corrected for multiple hypothesis testing).

## Reagents

Yeast strain construction

The strains used in this study are listed in Table 1. Gene deletions were constructed by transforming PCR products amplified from plasmids containing different deletion cassettes: pFA6a-kanMX for kanMX, pAC372 for natAC, and pUG66 for bleMX (Bähler et al., 1998; Gibney et al., 2015). Primers were designed with 40 flanking base pairs identical to the upstream and downstream region of genes to be deleted by homologous recombination. All gene deletions were made by transformation into a diploid to get a heterozygote, which was confirmed by PCR and then dissected to get *MAT* ***a*** and *MATα* segregants. Combinatorial gene deletion strains were made by mating, sporulating, and tetrad dissection.

Table 1. Strains used in this study

**Table:** 

Strain identifier	Names in text	Genotype/description	Reference
DBY12000	WT (haploid)	*MAT* ***a*** *HAP1* ^+^	See below ^a^
DBY12007	WT (diploid)	*HAP1* ^+^ / *HAP1* ^+^	
DBY12134	*tps1Δ*	*MAT* ***a*** *tps1Δ::kanMX*	this study
DBY12509	*hxk2Δ*	*MAT* ***a*** *hxk2Δ::bleMX*	
DBY12511	*hxk2Δ* *tps1Δ*	*MAT* ***a*** *hxk2Δ::bleMX tps1Δ::natAC*	
DBY12513	*grr1Δ*	*MAT* ***a*** *grr1Δ::bleMX*	
DBY12516	*grr1Δ* *tps1Δ*	*MAT* ***a*** *grr1Δ::bleMX tps1Δ::natAC*	
DBY12583	*tps1Δ* / *tps1Δ*	*tps1Δ::kanMX/tps1Δ::kanMX*	
DBY12584	*hxk2Δ/hxk2Δ*	*hxk2Δ::bleMX/hxk2Δ::bleMX*	
DBY12585	*hxk2Δ* / *hxk2Δ* *tps1Δ* / *tps1Δ*	*hxk2Δ::bleMX/hxk2Δ::bleMX* *tps1Δ::natAC/tps1Δ::natAC*	

a – All DBY12000-series strains are *HAP1* -repaired, *GAL+* , prototrophic derivatives of S288C. The details for constructing DBY12000 are found in (Hickman & Winston, 2007) . b – *natAC* refers to a version of the *natMX* dominant drug resistance marker cassette that contains a yeast codon-optimized *nat ^r^* gene.

## References

[R1] Bähler Jürg, Wu Jian-Qiu, Longtine Mark S., Shah Nirav G., Mckenzie III Amos, Steever Alexander B., Wach Achim, Philippsen Peter, Pringle John R. (1998). Heterologous modules for efficient and versatile PCR-based gene targeting inSchizosaccharomyces pombe. Yeast.

[R2] Bailey Richard B., Woodword Anne (1984). Isolation and characterization of a pleiotropic glucose repression resistant mutant of Saccharomyces cerevisiae. Molecular and General Genetics MGG.

[R3] Blázquez Miguel A., Gancedo Carlos (1995). Mode of action of the gcr9 and cat3 mutations in restoring the ability of Saccharomyces cerevisiae tps1 mutants to grow on glucose. Molecular and General Genetics MGG.

[R4] Blázquez Miguel A., Gancedo Carlos (1995). Mode of action of the gcr9 and cat3 mutations in restoring the ability of Saccharomyces cerevisiae tps1 mutants to grow on glucose. Molecular and General Genetics MGG.

[R5] Blázquez Miguel A., Lagunas Rosario, Gancedo Carlos, Gancedo Juana M. (1993). Trehalose-6-phosphate, a new regulator of yeast glycolysis that inhibits hexokinases. FEBS Letters.

[R6] Bolger Anthony M., Lohse Marc, Usadel Bjoern (2014). Trimmomatic: a flexible trimmer for Illumina sequence data. Bioinformatics.

[R7] Bonini Beatriz M., Van Dijck Patrick, Thevelein Johan M. (2003). Uncoupling of the glucose growth defect and the deregulation of glycolysis in Saccharomyces cerevisiae tps1 mutants expressing trehalose-6-phosphate-insensitive hexokinase from Schizosaccharomyces pombe. Biochimica et Biophysica Acta (BBA) - Bioenergetics.

[R8] Bonini BM, Van Vaeck C, Larsson C, Gustafsson L, Ma P, Winderickx J, Van Dijck P, Thevelein JM. 2000. Expression of escherichia coli otsA in a Saccharomyces cerevisiae tps1 mutant restores trehalose 6-phosphate levels and partly restores growth and fermentation with glucose and control of glucose influx into glycolysis. Biochem J 350 Pt 1: 261-8. PMC1221250 10926852

[R9] Chen Anqi, Gibney Patrick A. (2022). Intracellular trehalose accumulation via the Agt1 transporter promotes freeze–thaw tolerance in Saccharomyces cerevisiae. Journal of Applied Microbiology.

[R10] Chen Anqi, Vargas-Smith Jeremy, Tapia Hugo, Gibney Patrick A (2022). Characterizing phenotypic diversity of trehalose biosynthesis mutants in multiple wild strains of
*Saccharomyces cerevisiae*. G3 Genes|Genomes|Genetics.

[R11] Conklin Douglas S., Kung Ching, Culbertson Michael R. (1993). The
*COT2*
Gene Is Required for Glucose-Dependent Divalent Cation Transport in
*Saccharomyces cerevisiae*. Molecular and Cellular Biology.

[R12] De Silva-Udawatta Mihiri N., Cannon John F. (2001). Roles of trehalose phosphate synthase in yeast glycogen metabolism and sporulation. Molecular Microbiology.

[R13] Deroover Sofie, Ghillebert Ruben, Broeckx Tom, Winderickx Joris, Rolland Filip (2016). Trehalose-6-phosphate synthesis controls yeast gluconeogenesis downstream and independent of SNF1. FEMS Yeast Research.

[R14] Elbein A. D. (2003). New insights on trehalose: a multifunctional molecule. Glycobiology.

[R15] Eleutherio Elis C.A., Araujo Pedro S., Panek Anita D. (1993). Protective Role of Trehalose during Heat Stress in Saccharomyces cerevisiae. Cryobiology.

[R16] Erickson J R, Johnston M (1994). Suppressors reveal two classes of glucose repression genes in the yeast Saccharomyces cerevisiae.. Genetics.

[R17] Flick Jeffrey S., Johnston Mark (1991). *GRR1*
of
*Saccharomyces cerevisiae*
Is Required for Glucose Repression and Encodes a Protein with Leucine-Rich Repeats. Molecular and Cellular Biology.

[R18] GANCEDO C, FLORES C (2004). The importance of a functional trehalose biosynthetic pathway for the life of yeasts and fungi. FEMS Yeast Research.

[R19] Gibney Patrick A., Chen Anqi, Schieler Ariel, Chen Jonathan C., Xu Yifan, Hendrickson David G., McIsaac R. Scott, Rabinowitz Joshua D., Botstein David (2020). A tps1Δ persister-like state in Saccharomyces cerevisiae is regulated by MKT1. PLOS ONE.

[R20] Gibney Patrick A., Schieler Ariel, Chen Jonathan C., Rabinowitz Joshua D., Botstein David (2015). Characterizing the in vivo role of trehalose in
*Saccharomyces cerevisiae*
using the
*AGT1*
transporter. Proceedings of the National Academy of Sciences.

[R21] González M. Isabel, Blázquez Miguel A., Gancedo Carlos, Stucka Rolf, Feldmann Horst (1992). Molecular cloning ofCIF1, a yeast gene necessary for growth on glucose. Yeast.

[R22] 1991. Guide to yeast genetics and molecular biology. Methods Enzymol 194: 1-863. 2005781

[R23] Hickman Mark J., Winston Fred (2007). Heme Levels Switch the Function of Hap1 of
*Saccharomyces cerevisiae*
between Transcriptional Activator and Transcriptional Repressor. Molecular and Cellular Biology.

[R24] Hohmann Stefan, Bell Walter, NevesA Maria Jose, Valckx Dirk, Thevelein Johan M. (1996). Evidence for trehalose-6-phosphate-dependent and -independent mechanisms in the control of sugar influx into yeast glycolysis. Molecular Microbiology.

[R25] Hohmann Stefan, Neves Maria José, de Koning Wim, Alijo Rafael, Ramos José, Thevelein Johan M. (1993). The growth and signalling defects of the ggs1 (fdp1/byp1) deletion mutant on glucose are suppressed by a deletion of the gene encoding hexokinase PII. Current Genetics.

[R26] Hohmann Stefan, Winderickx Joris, de Winde Johannes H., Valckx Dirk, Cobbaert Philip, Luyten Kattie, de Meirsman Catherine, Ramos José, Thevelein Johan M. (1999). Novel alleles of yeast hexokinase PII with distinct effects on catalytic activity and catabolite repression of SUC2. Microbiology.

[R27] Hottiger Thomas, Boller Thomas, Wiemken Andres (1987). Rapid changes of heat and desiccation tolerance correlated with changes of trehalose content in
*Saccharomyces cerevisiae*
cells subjected to temperature shifts. FEBS Letters.

[R28] Hottiger Thomas, Boller Thomas, Wiemken Andres (1989). Correlation of trenalose content and heat resistance in yeast mutants altered in the RAS/adenylate cyclase pathway: is trehalose a thermoprotectant?. FEBS Letters.

[R29] Kim Jeong-Ho, Roy Adhiraj, Jouandot David, Cho Kyu Hong (2013). The glucose signaling network in yeast. Biochimica et Biophysica Acta (BBA) - General Subjects.

[R30] Li F. N. (1997). Grr1 of Saccharomyces cerevisiae is connected to the ubiquitin proteolysis machinery through Skp1: coupling glucose sensing to gene expression and the cell cycle. The EMBO Journal.

[R31] Li, H. (2013). Aligning sequence reads, clone sequences and assembly contigs with BWA-MEM. *ArXiv* , *13033997* , 1–3.

[R32] Liu Yanjie, Wood N. Ezgi, Marchand Ashley J., Arguello‐Miranda Orlando, Doncic Andreas (2020). Functional interrelationships between carbohydrate and lipid storage, and mitochondrial activity during sporulation in
*Saccharomyces cerevisiae*. Yeast.

[R33] Moreno F., Ahuatzi D., Riera A., Palomino C.A., Herrero P. (2005). Glucose sensing through the Hxk2-dependent signalling pathway. Biochemical Society Transactions.

[R34] Navon Gil, Shulman Robert G., Yamane Tetsuo, Eccleshall T. Ross, Lam Keng-Bon, Baronofsky Jerald J., Marmur Julius (1979). Phosphorus-31 nuclear magnetic resonance studies of wild-type and glycolytic pathway mutants of Saccharomyces cerevisiae. Biochemistry.

[R35] Özcan Sabire, Johnston Mark (1995). Three Different Regulatory Mechanisms Enable Yeast Hexose Transporter (
*HXT*
) Genes To Be Induced by Different Levels of Glucose. Molecular and Cellular Biology.

[R36] Petri, J. R. (1887). Eine kleine Modification des Koch’schen Plattenverfahrens (A minor modification of the pating technique of Koch). *Centralblatt Für Bacteriologie Und Parasitenkunde* , *1* , 279–280.

[R37] Robinson James T, Thorvaldsdóttir Helga, Winckler Wendy, Guttman Mitchell, Lander Eric S, Getz Gad, Mesirov Jill P (2011). Integrative genomics viewer. Nature Biotechnology.

[R38] Thevelein Johan M., Hohmann Stefan (1995). Trehalose synthase: guard to the gate of glycolysis in yeast?. Trends in Biochemical Sciences.

[R39] Vallier L G, Carlson M (1991). New SNF genes, GAL11 and GRR1 affect SUC2 expression in Saccharomyces cerevisiae.. Genetics.

[R40] Vallier L G, Coons D, Bisson L F, Carlson M (1994). Altered regulatory responses to glucose are associated with a glucose transport defect in grr1 mutants of Saccharomyces cerevisiae.. Genetics.

[R41] Aelst Linda, Hohmann Stefan, Bulaya Botchaka, Koning Wim, Sierkstra Laurens, Neves Maria José, Luyten Kattie, Alijo Rafael, Ramos José, Coccetti Paola, Martegani Enzo, Magalhães-Rocha Neuza Maria, Brandão Rogelio Lopes, Dijck Patrick, Vanhalewyn Mieke, Durnez Peter, Thevelein Johan M. (1993). Molecular cloning of a gene involved in glucose sensing in the yeast Saccharomyces cerevisiae. Molecular Microbiology.

[R42] van Heerden Johan H., Wortel Meike T., Bruggeman Frank J., Heijnen Joseph J., Bollen Yves J. M., Planqué Robert, Hulshof Josephus, O’Toole Tom G., Wahl S. Aljoscha, Teusink Bas (2014). Lost in Transition: Start-Up of Glycolysis Yields Subpopulations of Nongrowing Cells. Science.

[R43] VAN VAECK Christophe, WERA Stefaan, VAN DIJCK Patrick, THEVELEIN Johan M. (2000). Analysis and modification of trehalose 6-phosphate levels in the yeast Saccharomyces cerevisiae with the use of Bacillus subtilis phosphotrehalase. Biochemical Journal.

[R44] Vega Montserrat, Riera Alberto, Fernández-Cid Alejandra, Herrero Pilar, Moreno Fernando (2016). Hexokinase 2 Is an Intracellular Glucose Sensor of Yeast Cells That Maintains the Structure and Activity of Mig1 Protein Repressor Complex. Journal of Biological Chemistry.

[R45] Vicente Rebeca L., Spina Lucie, Gómez Jose P.L., Dejean Sebastien, Parrou Jean-Luc, François Jean Marie (2018). Trehalose-6-phosphate promotes fermentation and glucose repression in Saccharomyces cerevisiae. Microbial Cell.

[R46] Walther Thomas, Mtimet Narjes, Alkim Ceren, Vax Amélie, Loret Marie-Odile, Ullah Azmat, Gancedo Carlos, Smits Gertien J., François Jean Marie (2013). Metabolic phenotypes of
*Saccharomyces cerevisiae*
mutants with altered trehalose 6-phosphate dynamics. Biochemical Journal.

